# Assessing Potential Risks of Future Redo Transcatheter Aortic Valve Replacement in Asian Patients

**DOI:** 10.1016/j.jacasi.2023.09.004

**Published:** 2023-10-31

**Authors:** Norihisa Miyawaki, Kenichi Ishizu, Shinichi Shirai, Kenji Nakano, Tadatomo Fukushima, Euihong Ko, Yasuo Tsuru, Hiroaki Tashiro, Miho Nakamura, Hiroyuki Tabata, Toru Morofuji, Takashi Morinaga, Masaomi Hayashi, Akihiro Isotani, Nobuhisa Ohno, Shinichi Kakumoto, Kenji Ando

**Affiliations:** a Department of Cardiology, Kokura Memorial Hospital, Kitakyushu, Japan; b Department of Cardiovascular Surgery, Kokura Memorial Hospital, Kitakyushu, Japan; c Department of Anesthesiology, Kokura Memorial Hospital, Kitakyushu, Japan

**Keywords:** aortic stenosis, computed tomography, sinus sequestration, structural heart disease, transcatheter aortic valve replacement

## Abstract

**Background:**

In the Asian cohort, data are limited on the risk for coronary obstruction due to sinus of Valsalva (SOV) sequestration in redo transcatheter aortic valve replacement (TAVR) procedures.

**Objectives:**

The aim of this study was to assess the potential risk for coronary obstruction in simulated redo TAVR in Asian patients.

**Methods:**

Post-TAVR computed tomographic data from 788 patients who received balloon-expandable (BE) SAPIEN 3 transcatheter aortic valves (TAVs) and 334 patients who received self-expanding (SE) Evolut R or Evolut PRO TAVs were analyzed. The risk for coronary obstruction due to SOV sequestration in redo TAVR, defined as the TAV commissure level above the sinotubular junction (STJ) and a TAV-to-STJ distance <2.0 mm in each coronary sinus, was retrospectively evaluated.

**Results:**

The potential risks for coronary obstruction due to SOV sequestration at 1 or both coronary arteries were identified in 52.1% of the BE TAV group and 71.3% of the SE TAV group (*P* < 0.001). After adjusting for multiple covariates, STJ diameter, STJ height, TAV oversizing degree by area, and implantation depth were independently associated with SOV sequestration risk in the BE TAV group, whereas STJ diameter and implantation depth were independently associated with SOV sequestration risk in the SE TAV group.

**Conclusions:**

Coronary obstruction due to SOV sequestration in redo TAVR may occur in a substantial number of Asian patients. This finding suggests the importance of considering the structural feasibility of future redo TAVR when implanting the first TAV, especially in Asian patients with long life expectancy.

Transcatheter aortic valve (TAV) degeneration will become increasingly common with the expansion of the indication for TAV replacement (TAVR) to low-risk younger patients with longer life expectancy.^1^, ^2^, ^3^, ^4^ Although data regarding the long-term durability of TAVs are scarce, structural TAV degeneration occurs over time, as with surgical aortic bioprostheses, which may eventually require invasive interventions.^5^^,^^6^ Treatment of structural TAV degeneration by the implantation of a second TAV is considered feasible; however, the risk for coronary artery obstruction caused by sinus of Valsalva (SOV) sequestration is a serious concern. Theoretically, the second TAV will tilt up and pin open the leaflets of the first TAV, thereby creating a tube graft as tall as the commissural posts. Therefore, if the commissural posts of the first TAV are located above the sinotubular junction (STJ), future redo TAVR (ie, TAV-in-TAV) may not be possible, because the displaced leaflets of the first TAV may sequester the SOV, impairing blood flow to the coronary artery. Previous studies among Europeans and Americans reported the potential risk for SOV sequestration in future redo TAVR at 2.0% to 13.1% for balloon-expandable valves^7^^,^^8^ and 23.5% to 45.5% for supra-annular self-expanding (SE) valves.^7^^,^^9^ However, no data have been established on the risk among Asians with smaller aortic valve complexes, as well as smaller body sizes.^10^, ^11^, ^12^, ^13^ Thus, we investigated the prevalence and predictors of the potential risk for coronary obstruction caused by future redo TAVR simulation in Asian patients of Japanese ethnicity by analyzing the post-TAVR computed tomographic (CT) data from a large cohort of patients undergoing TAVR.

## Methods

### Study population and design

**Figure 1:**
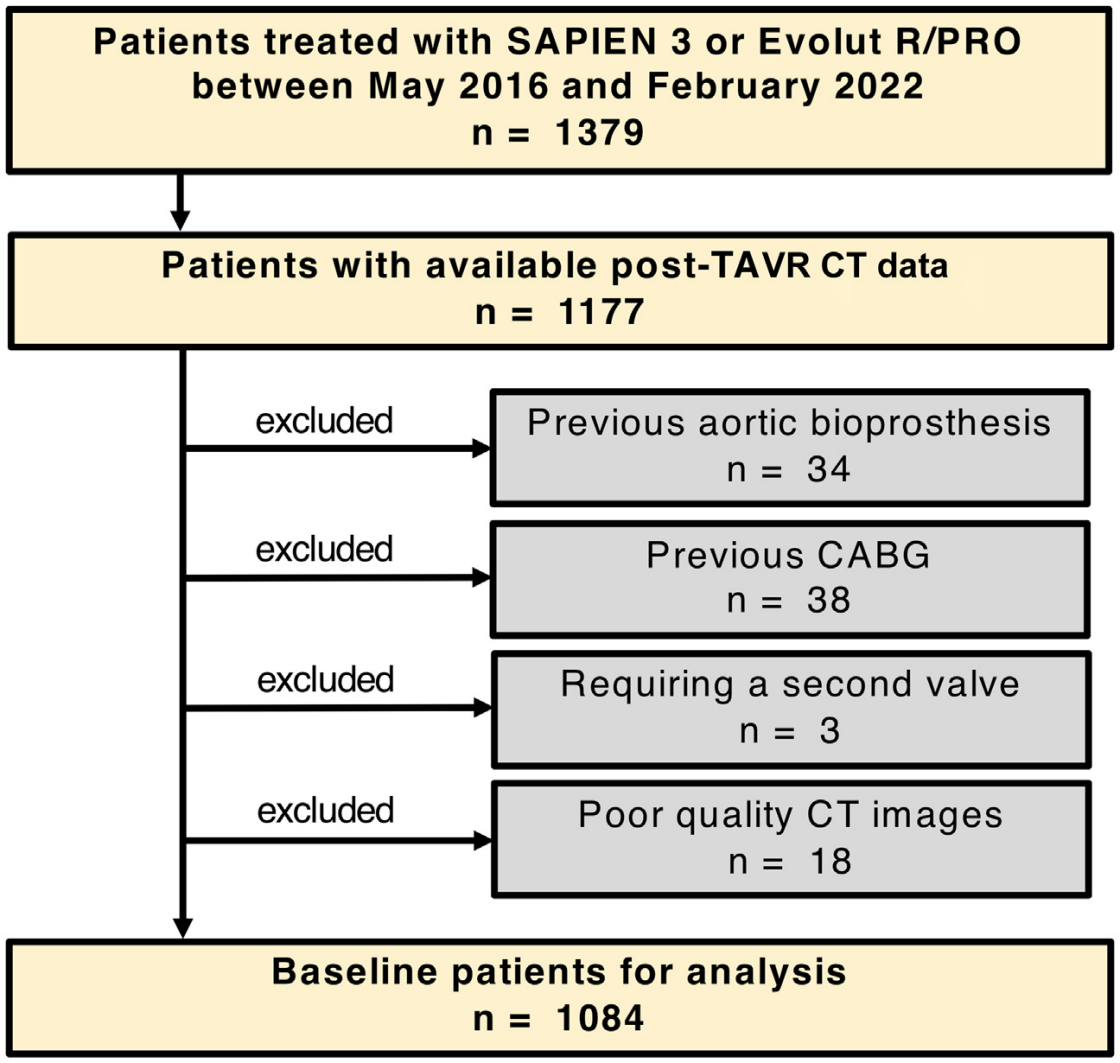
Study Workflow Flowchart providing information about the included and excluded patients. CABG = coronary artery bypass grafting; CT = computed tomographic; TAVR = transcatheter aortic valve replacement.

From May 2016 to February 2022, 1,379 consecutive patients undergoing TAVR with the BE SAPIEN 3 (Edwards Lifesciences) TAV or the supra-annular SE Evolut R or Evolut PRO (Medtronic) TAV at Kokura Memorial Hospital were prospectively included in an institutional database. All patients were considered ineligible or at high risk for surgical aortic valve replacement via consensus of the heart team, on the basis of not only surgical risk scores but also other factors, including age, frailty, and preoperative state for noncardiac surgery. Pre-TAVR electrocardiographically gated CT imaging was performed in all patients regardless of renal function, and post-TAVR electrocardiographically gated CT was routinely performed to assess the positional relation of the implanted prosthesis and the surrounding structures, including the coronary arteries and STJ, unless renal function precluded contrast administration. After excluding patients with previous aortic bioprosthesis, previous coronary artery bypass grafting, need for a second valve, or poor CT imaging quality from 1,177 patients with both pre- and post-TAVR CT studies, a total of 1,084 patients were included in the final analysis (Figure 1). The study conformed to the principles outlined in the Declaration of Helsinki and was approved by the Institutional Review Board of the Kokura Memorial Hospital Clinical Research Ethics Committee. Written informed consent was obtained from all patients before the TAVR procedure.

### CT acquisition protocol, image analysis, and definition

Pre- and post-TAVR electrocardiographically gated CT scans were performed using a 256-row system (Revolution CT, GE Healthcare) with a slice thickness of 0.625 mm and 25 to 70 mL intravenously administered contrast agent (Oypalomin 350, Fuji Pharma). The tube voltage and current were modified according to the patient’s body size. Image acquisition was, for the most part, performed with retrospective electrocardiographic gating. All images were reconstructed with a slice thickness of 0.625 mm and 50% slice overlap.

Offline analyses of CT Digital Imaging and Communications in Medicine data were performed using 3mensio Valves software version 7.0 or 8.0 (Pie Medical Imaging). As per the guideline, aortic annulus dimensions were measured in midsystole, while SOV and STJ were measured in diastole using pre-TAVR CT.^14^ The STJ height and coronary height were measured from the annular plane to the lowest point of the STJ and to the inferior border of each coronary ostium in a stretched multiplanar image, respectively.

**Figure 2:**
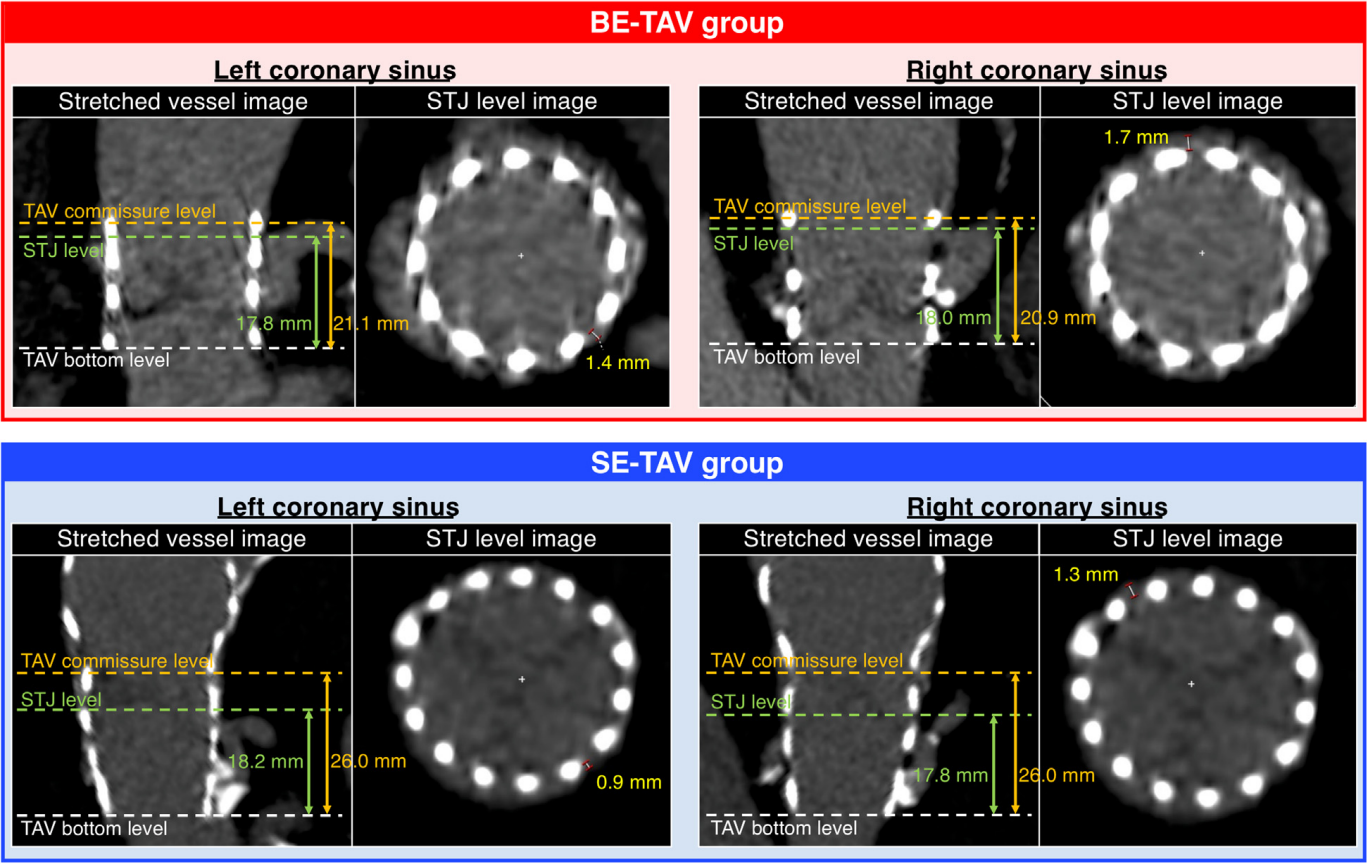
Representative Computed Tomographic Images With Coronary Obstruction Risk Representative computed tomographic images with potential sinus of Valsalva sequestration risk in redo transcatheter aortic valve replacement in the balloon-expandable (BE) transcatheter aortic valve (TAV) group (top panels) and the self-expanding (SE) TAV group (bottom panels) are shown. In the long-axis image, the distance from the inflow of the TAV to the sinotubular junction (STJ) in each coronary sinus was measured. In patients with TAV commissure levels above the STJ, the horizontal distance from the TAV to the STJ was measured in the short-axis view.

All post-TAVR CT analyses were performed in diastole. Following the study of Ochiai et al,^7^ the long- and short-axis views of TAV were identified using the 3 orthogonal multiplanar reconstruction planes. In the long-axis image, the distance from the inflow of the TAV to the STJ in each coronary sinus was measured to assess the positional relationship between the TAV and STJ. In patients with TAV commissure levels above the STJ, the horizontal distance from the central blooming artifact of the TAV to the STJ (TAV-to-STJ distance) was measured in the short-axis view. The simulated implantation of a second TAV will pin open the first TAV leaflets and contribute to SOV sequestration with impaired coronary flow; therefore, as Ochiai et al^7^ proposed, we defined coronary obstruction risk in redo TAVR as: 1) a TAV commissure level located above the STJ; and 2) a TAV-to-STJ distance <2.0 mm in each coronary sinus (Central Illustration, Figure 2). In this regard, however, it is necessary to take into account that the TAV commissure plane is often not parallel with the STJ plane because of the angulated implantation of the TAV. Therefore, in the present study, we carefully scanned the long-axis images of each coronary cusp and considered that the first condition was satisfied only when the TAV commissure level was located above the STJ in any of the long-axis images. A 2.0-mm cutoff was chosen because a 6-F catheter has an outer diameter of approximately 2.0 mm, whereas we performed additional analyses for TAV-to-STJ distance using the following modified criteria with different cutoffs as a sensitivity analysis: 1) TAV-to-STJ distance <3.0 mm; and 2) TAV-to-STJ distance <1.0 mm.

**Central Illustration:**
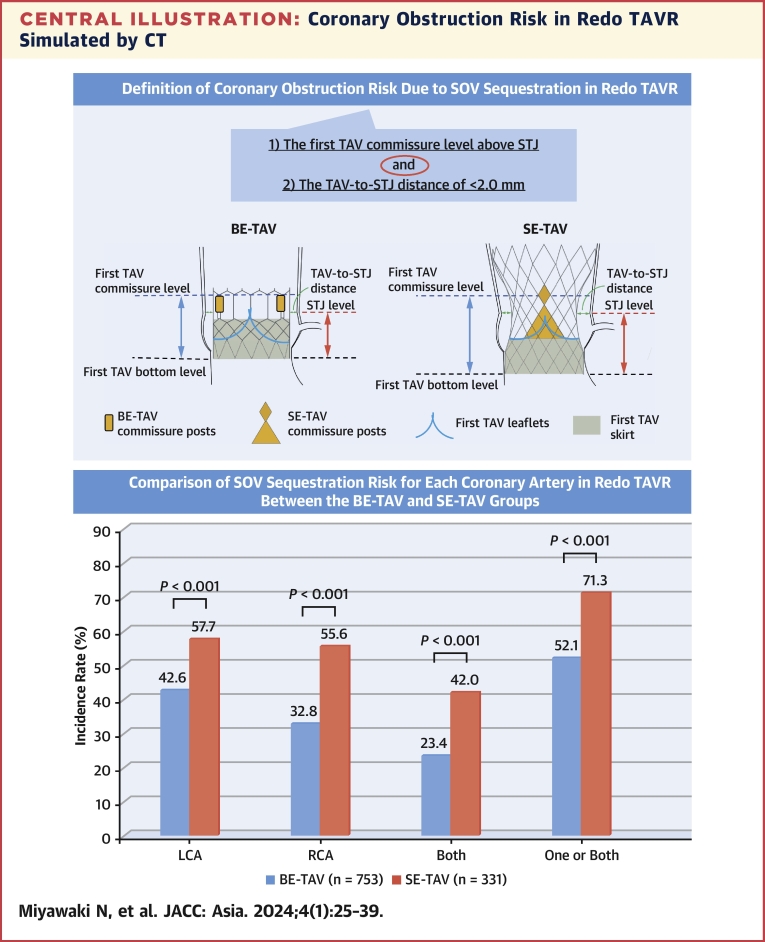
Coronary Obstruction Risk in Redo TAVR Simulated by CT (Top panel) Definition of coronary obstruction risk of sinus of Valsalva (SOV) sequestration in computed tomography (CT)–simulated redo transcatheter aortic valve replacement (TAVR). (Bottom panel) Incidence comparison of coronary obstruction risk caused by sequestering the SOV for each coronary artery in CT-simulated redo TAVR between the balloon-expandable (BE) transcatheter aortic valve (TAV) and self-expanding (SE) TAV groups. LCA = left coronary artery; RCA = right coronary artery; STJ = sinotubular junction.

For patients with potential risk for SOV sequestration during redo TAVR, the BASILICA (bioprosthetic or native aortic scallop intentional laceration to prevent iatrogenic coronary artery obstruction) technique can be an option to mitigate the risk.^15^ However, the BASILICA technique is considered unfeasible in patients with commissural malalignment of the first TAV. Therefore, the angle between the midpoint of each coronary ostium and the nearest commissural posts of TAV was also evaluated in the short-axis image. As previously reported, the first TAV commissure in front of each coronary was defined depending on the angle as follows: −5° to 5° in the BE TAV group and −24° to 24° for the 23-mm TAV and −36° to 36° for the 26-, 29-, and 34-mm TAVs in the SE TAV group (Supplemental Figure 1).^7^

Implantation depth of the TAV was expressed as the mean of the distances from the bottom of the coronary cusp to the proximal edge of the stent frame measured at each cusp.^16^ With respect to the BE TAV, TAV height was also measured on each cusp by using maximum-intensity projection,^16^ and we defined asymmetrical foreshortening of the TAV as (maximum TAV height/minimum TAV height − 1) × 100% of >10% (Supplemental Figure 2). Indeed, asymmetrical foreshortening may affect the degree to which TAV leaflets are tilted up during simulated redo TAVR, but because the effect is likely to be subtle and difficult to predict accurately, in our analysis, all leaflets of asymmetrically foreshortened TAVs were assumed to lift up to the commissure level as in usual cases.

All imaging studies were evaluated separately by 2 independent experienced readers (N.M. and K.I.), who were blinded to clinical information. In case of initial disagreement, consensus was reached for each patient after meticulous reanalysis. To evaluate intraobserver and interobserver variabilities for TAV-to-STJ distance, we also randomly selected a repeated measurement for a subset of 20 patients. Intraobserver and interobserver agreement was acceptable (intraclass correlation coefficients of 0.98 and 0.96, respectively).

### Statistical analysis

Categorical variables are expressed as number (percentage) and were compared using the chi-square test. Continuous variables are expressed as mean ± SD or median (Q1-Q3) and were compared using the independent Student’s *t*-test or Kruskal-Wallis test, depending on their distributions. The cumulative event rates were analyzed using Kaplan-Meier estimation, and differences were assessed using the log-rank test. To assess baseline structural factors associated with the risk for coronary obstruction due to SOV sequestration in simulated redo TAVR, multivariable logistic regression models were constructed. Candidate variables were selected a priori on the basis of assumptions of high theoretical or clinical relevance with reference to existing studies^7^, ^8^, ^9^ and included age, sex, body mass index, body surface area, annular area, valve size, STJ diameter, STJ height, degree of TAV oversizing, and implantation depth. We did not take the approach of entering any variables with *P* values <0.05 or <0.10 in the univariate analysis into the multivariate models, to avoid overfitting. ORs are reported with corresponding 95% CIs. Although to account for missing data values, we planned to use multiple imputation as a statistical plan, there was no missing value in the covariates constituting the multivariable models. Therefore, we conducted multivariable analyses for the outcome with complete cases. Receiver-operating characteristic curves were used to assess the predictive performance of these variables for coronary obstruction risk, and the best discriminatory thresholds were calculated by determining the Youden index. C statistics, indicating the areas under the receiver-operating characteristic curves, were compared between the variables using the method of DeLong et al.^17^

All statistical analyses were performed using JMP Pro 16.0.0 (SAS Institute) and R version 4.0.2 (R Foundation for Statistical Computing). A 2-tailed *P* value <0.05 was considered to indicate statistical significance.

## Results

Of the 1,084 patients eligible for analysis, 753 (69.5%) treated with BE TAVs and 331 (30.5%) with SE TAVs were identified. Baseline patient characteristics according to TAV type are shown in Supplemental Table 1. Of note, both kinds of TAVs were implanted “high,” in accordance with current clinical practice, albeit with smaller implantation depths in patients with BE TAVs than in those with SE TAVs (median 2.1 mm [Q1-Q3: 0.9-3.4 mm] vs 3.2 mm [Q1-Q3: 1.6-4.5 mm]; *P* < 0.001). Temporal downtrends of implantation depth were also observed in both groups (*P* < 0.001 for trend for both) (Supplemental Figure 3). With regard to the BE TAV group, asymmetrical foreshortening was observed in 79 patients (10.5%).

### Association between TAV commissure level and STJ height

TAV commissure level above the STJ was observed in 422 patients (56.0%) for the left coronary sinus (LCS) and in 266 patients (35.3%) for the right coronary sinus (RCS) in the BE TAV group, whereas it was observed in 297 patients (89.7%) for the LCS and in 290 patients (87.6%) for the RCS in the SE TAV group.

### TAV-to-STJ distance

In patients with TAV commissure levels located above the STJ, TAV-to-STJ distances were evaluated. In the BE TAV group, TAV-to-STJ distances <2.0 mm were observed in 321 patients (76.1%) for the LCS and 247 patients (92.9%) for the RCS. Conversely, TAV-to-STJ distances <2.0 mm were noted in 191 patients (64.3%) for the LCS and 184 patients (63.4%) for the RCS in the SE TAV group.

#### Coronary obstruction risk in redo TAVR simulated by CT imaging

**Figure 3:**
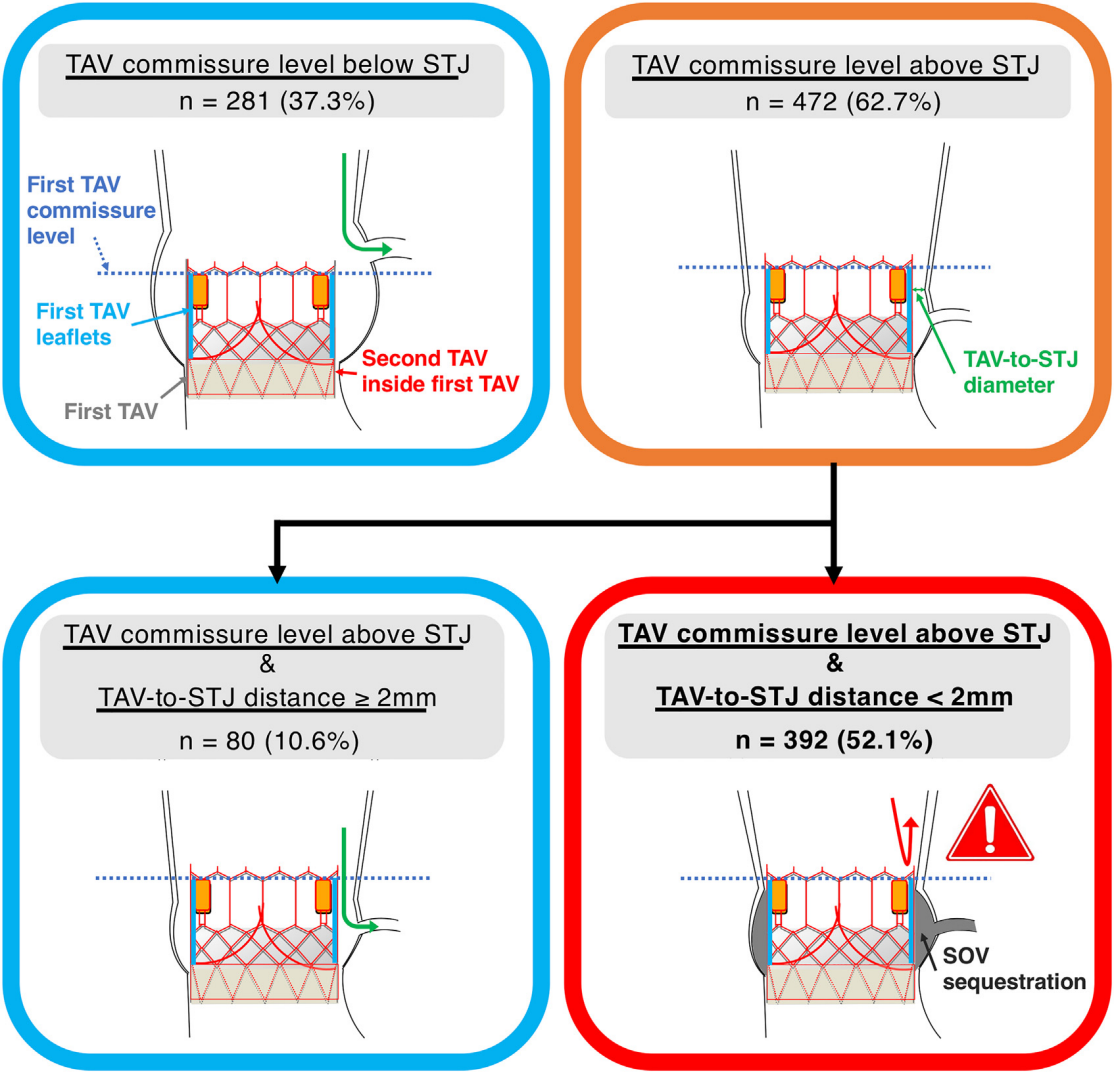
Feasibility of Future Redo TAV Replacement in the BE TAV Group In 472 patients with TAV commissure levels located above the STJ, TAV-to-STJ distances <2.0 mm were observed in 392 patients (83.1%) for 1 or both coronary sinuses in the BE TAV group. Abbreviations as in Figure 2.

**Figure 4:**
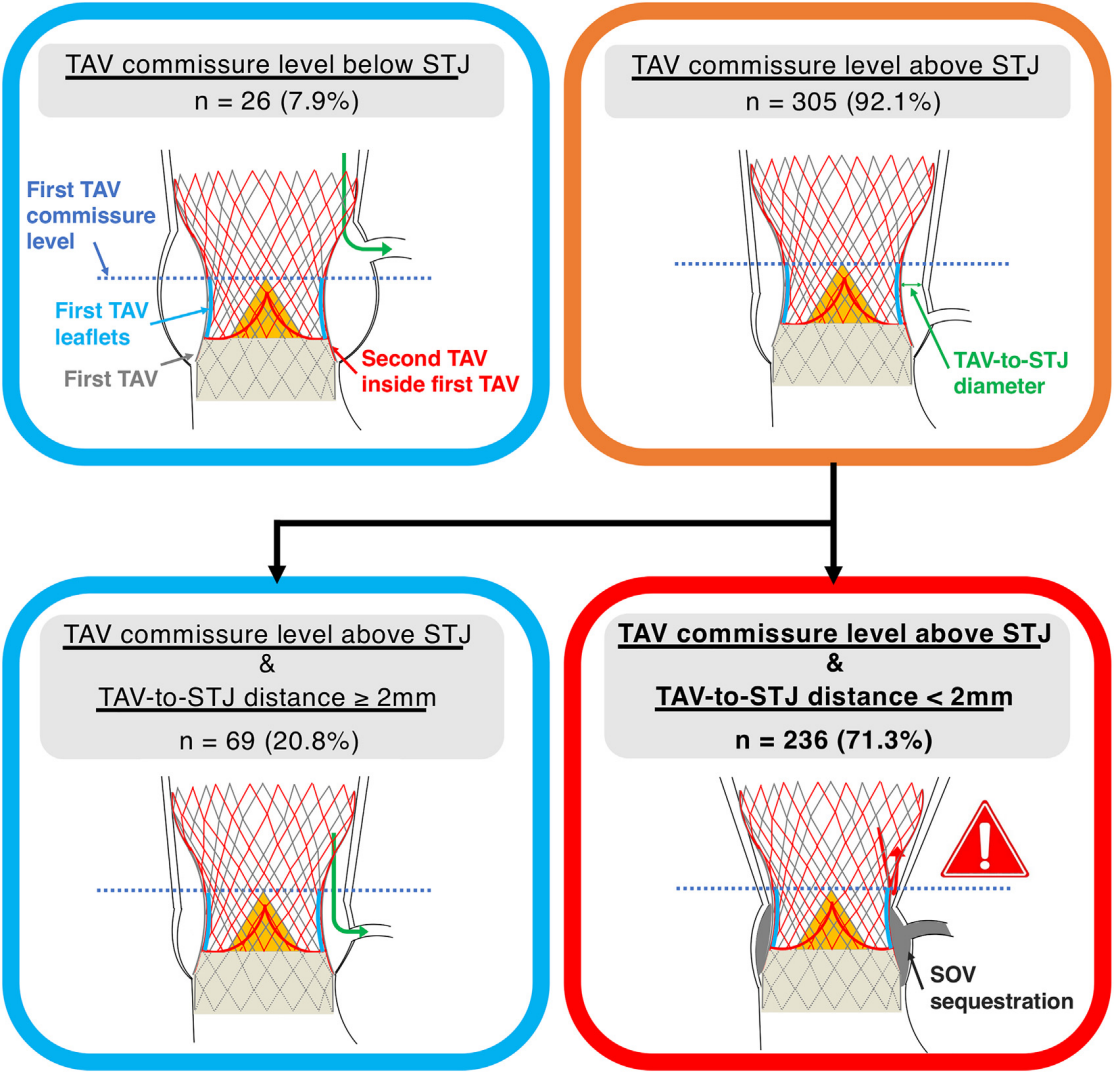
Feasibility of Future Redo TAV Replacement in the SE TAV Group In 305 patients with TAV commissure levels located above the STJ, TAV-to-STJ distances <2.0 mm were observed in 236 patients (77.4%) for 1 or both coronary sinuses in the SE TAV group. Abbreviations as in Figure 2.

**Figure 5:**
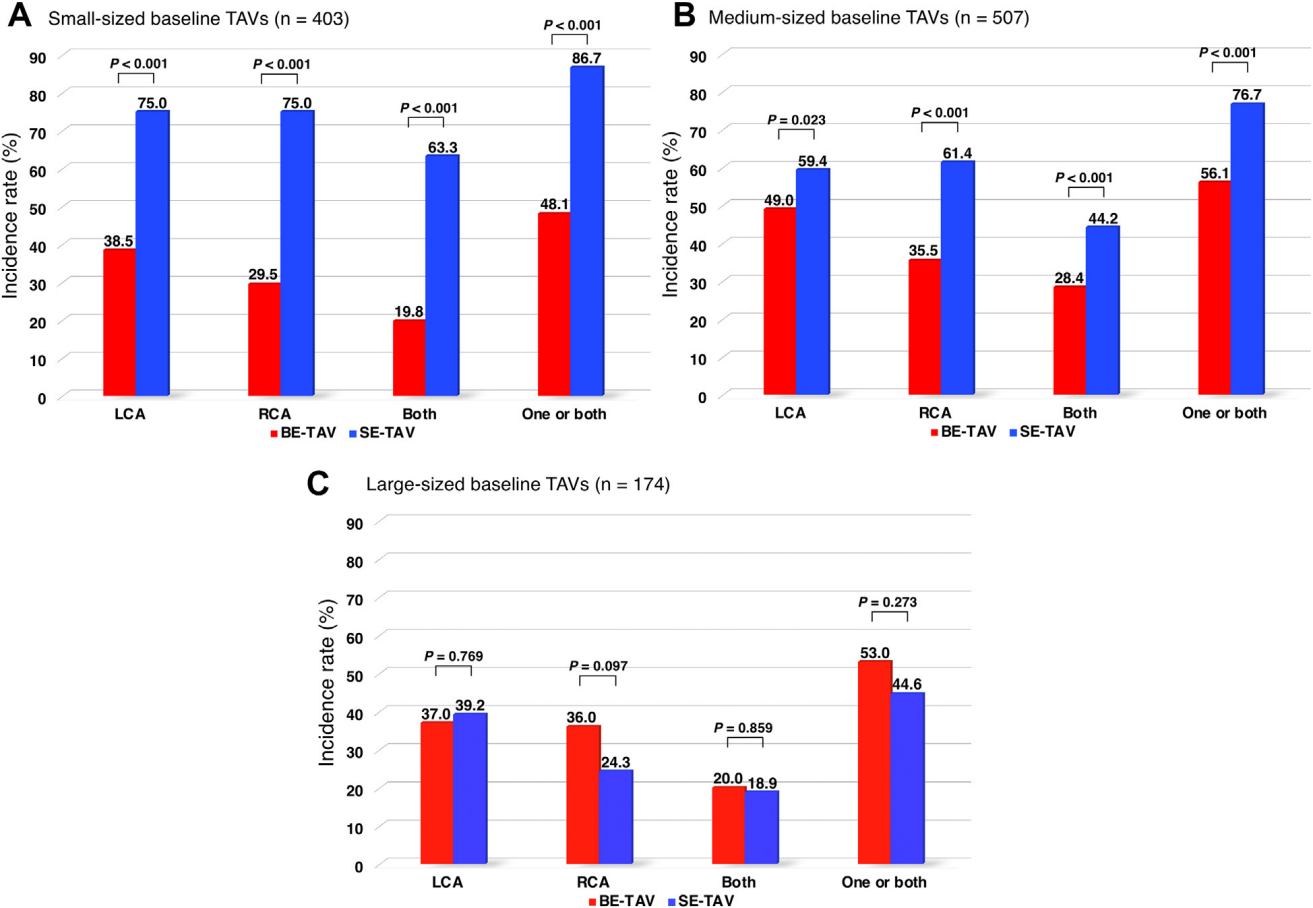
Coronary Obstruction Risk in Redo TAV Replacement by Baseline TAV Sizes (A) Small baseline TAVs (20- and 23-mm BE TAVs and 23-mm SE BAVs), (B) medium-sized baseline TAVs (26-mm BE TAVs and 26-mm SE TAVs), and (C) large baseline TAVs (29-mm BE TAVs and 29- and 34-mm SE TAVs). LCA = left coronary artery; RCA = right coronary artery; other abbreviations as in Figure 2.

CT simulation predicted that coronary obstruction by sequestering SOV might occur in 321 patients (42.6%) for the left coronary artery (LCA) and 247 patients (32.8%) for the right coronary artery (RCA) in the BE TAV group, whereas the risk was observed in 191 patients (57.7%) for the LCA and 184 patients (55.6%) for the RCA in the SE TAV group. In addition, obstruction risk of 1 or both coronary arteries was identified in 392 patients (52.1%) in the BE TAV group and 236 patients (71.3%) in the SE TAV group (*P* < 0.001) (Figures 3 and 4, Central Illustration). We performed additional analyses by trichotomizing patients according to baseline TAV size as follows: small (20- and 23-mm BE TAVs and 23-mm SE BAVs), medium (26-mm BE TAVs and 26-mm SE TAVs), and large (29-mm BE TAVs and 29- and 34-mm SE TAVs). In patients with baseline small or medium-sized TAVs, coronary obstruction by SOV sequestration might occur more frequently in the SE TAV group, whereas the risk was found to be comparable between the BE TAV and SE TAV groups in those with the baseline large TAVs (Figure 5).

Baseline characteristics according to obstruction risk in 1 or both coronary arteries in the BE TAV and SE TAV groups are shown in Tables 1 and 2, respectively. In the BE TAV group, STJ height, STJ diameter, mean SOV diameter, and coronary artery height were significantly smaller in patients who were at coronary obstruction risk than in control patients, albeit with comparable annular sizes between the groups. Although implanted TAV sizes did not differ between the 2 groups, a greater degree of TAV oversizing by area and a higher TAV position were observed in patients at coronary obstruction risk. Meanwhile, in the SE TAV group, any aortic root size, including annular size, left ventricular outflow tract area, STJ height, STJ diameter, mean SOV diameter, and coronary artery height, was smaller in patients at coronary obstruction risk than in control patients. Although implanted TAV sizes and depths were also smaller in patients who were at coronary obstruction risk, the degree of TAV oversizing by perimeter was similar between the 2 groups.

**Table 1:** Baseline Characteristics According to Coronary Obstruction Risk in the Balloon-Expandable TAV Group

	Total (N = 753)	Coronary Obstruction Risk		*P* Value
		Yes (n = 392)	No (n = 361)	
Demographics				
Age, y	85 (81-88)	84 (80-87)	85 (81-88)	0.143
Male	294 (39.0)	134 (34.2)	160 (44.3)	0.004
Height, cm	150.0 (144.9-159.0)	149.5 (144.3-157.7)	151.0 (145.0-160.0)	0.094
Weight, kg	51.2 (43.7-59.5)	52.0 (44.1-59.8)	50.6 (43.5-59.4)	0.430
Body mass index, kg/m^2^	22.4 (20-24.8)	22.8 (20.3-25.1)	21.8 (19.6-24.4)	0.011
Body surface area, m^2^	1.4 (1.3-1.6)	1.4 (1.3-1.6)	1.5 (1.3-1.6)	0.976
Clinical Frailty Scale score	3 (3-4)	3 (3-4)	3 (3-4)	0.100
NYHA functional class III/IV	280 (37.2)	127 (32.4)	153 (42.4)	0.005
STS-PROM score, %	5.1 (3.5-7.8)	5.0 (3.4-7.4)	5.2 (3.6-8.1)	0.044
Comorbidities				
Hypertension	614 (81.5)	328 (83.7)	286 (79.2)	0.116
Dyslipidemia	399 (53.0)	210 (53.6)	189 (52.4)	0.738
Diabetes mellitus	193 (24.3)	99 (25.3)	84 (23.3)	0.729
Atrial fibrillation	172 (22.8)	76 (19.4)	96 (26.6)	0.019
Coronary artery disease	299 (39.7)	153 (39.0)	146 (40.4)	0.692
Previous percutaneous coronary intervention	155 (20.6)	71 (18.1)	84 (23.3)	0.081
Previous valve surgery	5 (0.7)	2 (0.5)	3 (0.8)	0.588
Previous permanent pacemaker	43 (5.7)	13 (3.3)	30 (9.0)	0.008
Peripheral artery disease	64 (8.5)	29 (7.4)	35 (9.7)	0.259
Chronic obstructive pulmonary disease	58 (7.7)	25 (6.4)	33 (9.1)	0.155
Cerebrovascular disease	97 (12.9)	45 (11.5)	52 (14.4)	0.232
Active cancer	56 (7.4)	27 (6.9)	29 (8.0)	0.550
Blood tests				
Hemoglobin, g/dL	11.5 (10.2-12.6)	11.5 (10.4-12.6)	11.5 (10.1-12.6)	0.629
eGFR, mL/min/1.73 m^2^	52.7 (40.9-65.6)	54.2 (40.8-67.1)	51.6 (40.9-63.5)	0.239
Albumin, g/dL	3.7 (3.4-4.0)	3.8 (3.4-4.0)	3.7 (3.4-4.0)	0.173
Brain natriuretic peptide, pg/mL	135.3 (60.9-345.6)	111.4 (58.9-338.0)	163.1 (65.7-349.5)	0.055
Echocardiographic data				
Aortic valve area, cm^2^	0.72 (0.61-0.81)	0.70 (0.60-0.80)	0.73 (0.62-0.83)	0.004
Indexed aortic valve area, cm^2^/m^2^	0.50 (0.40-0.57)	0.50 (0.40-0.56)	0.50 (0.40-0.58)	0.009
Mean aortic gradient, mm Hg	40.7 (31.1-52.4)	42.7 (34.0-55.4)	38.6 (28.3-48.2)	<0.001
Left ventricular ejection fraction, %	62.2 (54.6-65.1)	62.7 (55.8-65.2)	61.5 (53.3-65.1)	0.063
Left ventricular end-diastolic diameter, mm	44.6 (40.8-48.6)	44.3 (40.8-47.8)	44.9 (40.9-49.3)	0.114
Aortic regurgitation moderate or greater	47 (6.2)	21 (5.4)	26 (7.2)	0.296
Mitral regurgitation moderate or greater	42 (5.6)	21 (5.4)	21 (5.8)	0.784
Tricuspid regurgitation moderate or greater	30 (4.0)	13 (3.3)	17 (4.7)	0.329
Systolic pulmonary arterial pressure, mm Hg	31.0 (26.0-37.0)	31.0 (27.0-36.1)	31.7 (25.0-37.8)	0.845
Pre-TAVR CT data				
Annular area, mm^2^	420.1 (373.4-481.0)	424.0 (373.6-472.7)	414.0 (373.0-489.1)	0.950
Annular perimeter, mm	73.2 (69.2-78.3)	73.5 (69.0-77.7)	73.1 (69.4-79.0)	0.820
LVOT area, mm^2^	408.9 (343.6-497.3)	413.9 (347.6-489.5)	404.5 (341.0-505.6)	0.718
STJ height, mm	19.0 (17.1-20.9)	18.3 (16.5-20.3)	19.6 (17.7-21.9)	<0.001
STJ diameter, mm	25.8 (24.1-28.1)	25.1 (23.4-26.7)	26.8 (24.7-29.1)	<0.001
Mean SOV diameter, mm	30.0 (28.3-32.5)	29.5 (27.8-31.4)	31.0 (28.8-33.4)	<0.001
Ascending aortic diameter at 40 mm, mm	32.8 (30.8-34.6)	32.5 (30.8-34.3)	33.2 (31.0-35.3)	0.003
Left coronary artery height, mm	13.8 (12.4-15.2)	13.3 (12.0-14.5)	14.2 (12.9-15.7)	<0.001
Right coronary artery height, mm	15.5 (13.5-17.7)	15.0 (13.0-17.0)	16.3 (14.0-18.5)	<0.001
Bicuspid valve	32 (4.3)	11 (2.8)	21 (5.8)	0.040
Procedural characteristics				
TAV size				
20 mm	17 (2.3)	7 (1.8)	10 (2.8)	0.204
23 mm	326 (43.3)	158 (40.3)	168 (46.5)	
26 mm	310 (41.2)	174 (44.4)	136 (37.7)	
29 mm	100 (13.3)	53 (13.5)	47 (13.0)	
Oversizing by area, %	12.3 (4.9-19.8)	14.4 (7.4-20.5)	9.7 (3.6-17.9)	<0.001
Post-TAVR CT data				
Implantation depth, mm	2.1 (0.9-3.4)	2.0 (0.8-3.1)	2.3 (1.1-3.8)	0.002

Values are median (Q1-Q3) or n (%).

CT = computed tomographic; eGFR = estimated glomerular filtration rate; LVOT = left ventricular outflow tract; SOV = sinus of Valsalva; STJ = sinotubular junction; STS-PROM = Society of Thoracic Surgeons Predicted Risk of Mortality; TAV = transcatheter aortic valve; TAVR = transcatheter aortic valve replacement.

**Table 2:** Baseline Characteristics According to Coronary Obstruction Risk in the Self-Expanding TAV Group

	Total (N = 331)	Coronary Obstruction Risk		*P* Value
		Yes (n = 236)	No (n = 95)	
Demographics				
Age, y	85 (81-88)	85 (82-88)	86 (81-89)	0.948
Male	78 (23.6%)	32 (13.6%)	46 (48.4%)	<0.001
Height, cm	148.0 (143.0-154.4)	146.7 (142.0-152.0)	151.8 (145.3-161.0)	<0.001
Weight, kg	47.3 (41.9-54.9)	46.2 (40.9-54.0)	50.1 (44.6-58.4)	0.001
Body mass index, kg/m^2^	21.6 (19.5-24.3)	21.5 (19.4-24.3)	21.7 (19.9-24.4)	0.471
Body surface area, m^2^	1.4 (1.3-1.5)	1.4 (1.3-1.5)	1.4 (1.4-1.6)	<0.001
Clinical Frailty Scale score	3 (3-4)	3 (3-4)	3 (3-4)	0.276
NYHA functional class III/IV	102 (30.8)	77 (32.6)	25 (26.3)	0.256
STS-PROM score, %	5.1 (3.7-7.4)	5.1 (4.0-7.7)	4.8 (3.1-6.8)	0.022
Comorbidities				
Hypertension	266 (80.4)	188 (79.7)	78 (82.1)	0.610
Dyslipidemia	164 (49.6)	125 (53.0)	39 (41.1)	0.049
Diabetes mellitus	67 (20.2)	49 (20.8)	18 (19.0)	0.931
Atrial fibrillation	57 (17.2)	39 (16.5)	18 (19.0)	0.600
Coronary artery disease	110 (33.2)	81 (34.3)	29 (30.5)	0.505
Previous percutaneous coronary intervention	52 (15.7)	38 (16.1)	14 (14.7)	0.756
Previous valve surgery	0 (0)	0 (0)	0 (0)	-
Previous permanent pacemaker	11 (3.3)	10 (4.2)	1 (1.1)	0.253
Peripheral artery disease	14 (4.2)	11 (4.7)	3 (3.2)	0.527
Chronic obstructive pulmonary disease	17 (5.1)	6 (2.5)	11 (11.6)	0.002
Cerebrovascular disease	33 (10.0)	14 (5.9)	19 (20.0)	<0.001
Active cancer	16 (4.8)	9 (3.8)	7 (7.4)	0.189
Blood tests				
Hemoglobin, g/dL	11.2 (10.1-12.2)	11.2 (10.0-12.2)	11.5 (10.3-12.3)	0.084
eGFR, mL/min/1.73 m^2^	54.4 (43.2-67.5)	53.8 (41.2-68.6)	56.5 (45.8-65.4)	0.615
Albumin, g/dL	3.8 (3.4-4.0)	3.8 (3.4-4.0)	3.8 (3.4-4.1)	0.679
Brain natriuretic peptide, pg/mL	146.8 (62.4-361.8)	126.0 (63.8-307.8)	220.9 (58.5-503.5)	0.035
Echocardiographic data				
Aortic valve area, cm^2^	0.63 (0.51-0.73)	0.63 (0.52-0.73)	0.63 (0.5-0.71)	0.530
Indexed aortic valve area, cm^2^/m^2^	0.44 (0.39-0.50)	0.46 (0.40-0.52)	0.40 (0.33-0.50)	0.005
Mean aortic gradient, mm Hg	53.9 (41.5-68.0)	52.9 (40.7-68.3)	56.5 (45.5-67.4)	0.350
Left ventricular ejection fraction, %	63.6 (59.5-66.0)	63.7 (59.9-66.0)	62.7 (58.2-65.7)	0.109
Left ventricular end-diastolic diameter, mm	42.5 (39.1-46.1)	41.7 (39.0-45.3)	44.8 (40.1-47.6)	<0.001
Aortic regurgitation moderate or greater	12 (3.6)	7 (3.0)	5 (5.3)	0.328
Mitral regurgitation moderate or greater	17 (5.1)	11 (4.7)	6 (6.3)	0.545
Tricuspid regurgitation moderate or greater	11 (3.3)	6 (2.5)	5 (5.3)	0.231
Systolic pulmonary arterial pressure, mm Hg	31.0 (26.0-37.8)	31.0 (26.0-37.0)	31.5 (25.0-39.3)	0.492
Pre-TAVR CT data				
Annular area, mm^2^	387.0 (345.5-432.5)	373.8 (339.9-413.0)	422.1 (371.3-476.3)	<0.001
Annular perimeter, mm	70.4 (66.3-74.7)	69.1 (65.5-72.6)	74.4 (69.3-78.6)	<0.001
LVOT area, mm^2^	364.0 (316.3-424.7)	354.7 (306.8-398.8)	403.6 (338.9-479.2)	<0.001
STJ height, mm	18.1 (16.4-19.7)	17.8 (16.0-19.2)	19.1 (17.1-21.7)	<0.001
STJ diameter, mm	24.4 (22.6-26.3)	23.5 (22.3-25.2)	27 (24.8-29.2)	<0.001
Mean SOV diameter, mm	28.8 (27.3-30.8)	28.1 (26.9-29.6)	31.4 (29.2-32.9)	<0.001
Ascending aortic diameter at 40 mm, mm	31.7 (30.1-33.5)	31.1 (29.5-32.7)	33.6 (31.6-35.9)	<0.001
Left coronary artery height, mm	13.5 (12.0-14.8)	13.3 (11.9-14.4)	14.2 (12.4-15.1)	0.004
Right coronary artery height, mm	14.6 (12.6-16.6)	14.2 (12.5-16.2)	15.1 (12.7-18.2)	0.005
Bicuspid valve	34 (10.3)	14 (5.9)	20 (21.1)	<0.001
Procedural characteristics				
TAV size				
23 mm	60 (18)	52 (22.0)	8 (8.4)	<0.001
26 mm	197 (59.5)	151 (64.0)	46 (48.4)	
29 mm	71 (21.5)	33 (14.0)	38 (40.0)	
34 mm	3 (0.9)	0 (0)	3 (3.2)	
Oversizing by perimeter, %	16.3 (12.8-20.8)	16.3 (13.1-20.8)	16.3 (12.3-20.8)	0.301
Post-TAVR CT data				
Implantation depth, mm	3.2 (1.6-4.5)	3.0 (1.5-4.4)	3.8 (2.0-5.3)	0.016

Values are median (Q1-Q3) or n (%).

Abbreviations as in Table 1.

We also performed sensitivity analyses using different cutoffs for TAV-to-STJ distance. Obstruction risk of 1 or both coronary arteries was identified in 430 patients (57.1%) in the BE TAV group and 286 patients (86.4%) in the SE TAV group (*P* < 0.001) according to the modified criteria (TAV-to-STJ distance <3.0 mm), while risk was identified in 292 patients (38.8%) in the BE TAV group and 192 patients (58.0%) in the SE TAV group (*P* < 0.001) according to the modified criteria (TAV-to-STJ distance <1.0 mm).

### Predictors of coronary obstruction in simulated redo TAVR

To identify predictors of coronary obstruction risk due to SOV sequestration in simulated redo TAVR, multivariable logistic regression models were constructed. After adjustment for age, sex, body mass index, body surface area, annular area, and valve size, STJ diameter (OR per 1.0-mm decrease: 1.53; 95% CI: 1.39-1.69; *P* < 0.001), STJ height (OR per-1.0 mm decrease: 1.33; 95% CI: 1.22-1.44; *P* < 0.001), TAV oversizing degree by area (OR per 5% increase: 1.52, 95% CI: 1.36-1.69; *P* < 0.001), and implantation depth (OR per 1.0-mm decrease: 1.45; 95% CI: 1.29-1.63; *P* < 0.001) were independently associated with coronary obstruction risk in the BE TAV group, whereas STJ diameter (OR per 1.0-mm decrease: 1.63; 95% CI: 1.39-1.91; *P* < 0.001) and implantation depth (OR per 1.0-mm decrease: 1.16; 95% CI: 1.02-1.33; *P* = 0.025) were independently associated with coronary obstruction risk in the SE TAV group (Table 3).

**Table 3:** Multivariable Analyses of Predictors of Coronary Obstruction in Redo TAV Replacement

	Multivariable Analysis					
	BE TAV Group			SE TAV Group		
	OR	95% CI	*P* Value	OR	95% CI	*P* Value
STJ diameter (per 1.0-mm decrease)	1.53	1.39-1.69	<0.001	1.63	1.39-1.91	<0.001
STJ height (per 1.0-mm decrease)	1.33	1.22-1.44	<0.001	1.00	0.88-1.14	0.982
Oversizing by area (per 5% increase)^a^	1.52	1.36-1.69	<0.001	—	—	—
Oversizing by perimeter (per 5% increase)^a^	—	—	—	1.06	0.52-2.18	0.871
Implantation depth (per 1.0-mm decrease)	1.45	1.29-1.63	<0.001	1.16	1.02-1.33	0.025

Adjusted by age, sex, body mass index, body surface area, annular area, and valve size.

BE = balloon-expandable; SE = self-expanding; other abbreviations as in Table 1.

a The degrees of TAV oversizing relative to annulus were assessed by area in the BE TAV group and by perimeter in the SE TAV group.

Comparative analysis assessing the discrimination values of these factors showed that STJ diameter was a better predictor of coronary obstruction risk than TAV oversizing ratio or implantation depth in both the BE TAV and SE TAV groups. However, the BE TAV group showed comparable predictive values between STJ diameter and STJ height, whereas STJ diameter yielded a higher predictive ability than STJ height in the SE TAV group (Supplemental Figure 4).

### TAV commissural malalignment with coronary arteries

Coronary-level analyses in patients with potential coronary obstruction risk due to SOV sequestration showed that the first TAV commissure in front of each coronary ostium was identified in 21 of 321 patients (6.5%) for the LCA and 13 of 247 patients (5.3%) for the RCA in the BE TAV group and in 86 of 191 patients (45.0%) for the LCA and 86 of 184 patients (46.7%) for the RCA in the SE TAV group.

## Discussion

The risk for coronary obstruction due to SOV sequestration in redo TAVR is not a new concept; however, this is the first study using a large prospective registry composed of Asian patients of Japanese ethnicity to evaluate how often redo TAVR in the first BE TAV and SE TAV might risk coronary obstruction. The major findings of this study are as follows: 1) redo TAVR simulated by post-TAVR CT imaging showed that coronary obstruction risk was identified in 52.1% patients in the BE TAV group and 71.3% patients in the SE TAV group; 2) after adjusting for multiple covariates, STJ diameter, STJ height, TAV oversizing degree by area, and implantation depth were independently associated with coronary obstruction risk in the BE-TAV group, whereas STJ diameter and implantation depth were independently associated with coronary obstruction risk in the SE TAV group; and 3) commissure malalignment of the first TAV might prevent a considerable portion of patients at risk for coronary obstruction from benefiting from the BASILICA technique, which is aimed at mitigating the coronary obstruction risk.

Coronary obstruction is a serious procedural complication of TAVR for native aortic valves or failed surgically implanted aortic bioprostheses that is associated with a high mortality rate.^18^ Data are scarce on coronary obstruction in redo TAVR; however, given the mechanism, a low success rate of coronary recanalization resulting in a higher mortality rate is easy to imagine. Unlike native aortic valves and failed surgically implanted aortic bioprostheses, the first valve leaflets in redo TAVR do not reach the coronary ostia, because of interruption by the stent frame of the first TAV. Instead, they may sequester the SOV by forming a tube graft as tall as the commissural posts. Bailout percutaneous coronary intervention for SOV sequestration is considered technically unfeasible; therefore, preprocedural anatomical assessment and meticulous CT simulation are essential for redo TAVR candidates. Indeed, a recent study from the Redo-TAVR registry demonstrated a low rate of coronary obstruction (0.9%) with redo TAVR.^19^ However, notably, the number of patients who were not indicated for redo TAVR because of concern for coronary obstruction was not captured.

Previous studies on simulated redo TAVR among Europeans and Americans have reported the potential risk for coronary obstruction because of SOV sequestration at 2.0% to 13.1% for the BE TAV^7^^,^^8^ and 23.5% to 45.5% for the SE TAV.^7^^,^^9^ In particular, the study of Ochiai et al,^7^ in which the definition of coronary obstruction risk and the methodology for CT measurements were consistent with those of our study, demonstrated the percentages to be 2.0% for the BE TAV and 45.2% for the SE TAV. Compared with that study, our analysis exclusively among Asians of Japanese ethnicity demonstrated a substantially higher rate of coronary obstruction risk in future redo TAVR, at 52.1% in the BE TAV group and 71.3% in the SE TAV group. Asian candidates for TAVR, including Japanese patients, are reported to have smaller aortic complexes and body size than Western patients.^10^, ^11^, ^12^, ^13^ From an anatomical perspective, patients with small and low STJs would have an increased risk for coronary obstruction. In fact, prior studies assessing the feasibility of redo TAVR among European and American patients demonstrated, without exception, larger aortic complexes that were accordingly treated with larger TAVs, compared with our study.^7^, ^8^, ^9^ Notably, however, the patients in our Japanese cohort actually presented with relatively smaller surrounding structures than the annulus. In particular, the tendency was stronger in the SE TAV group in our study because BE TAV implantation for patients with relatively small STJs was deemed at risk for ascending aortic dissection. In addition, the Evolut R and Evolut PRO TAV commissure height was 26 mm irrespective of TAV size; thus, several patients in the SE TAV group who had received smaller TAVs inevitably had lower STJ heights, which could increase the risk for SOV sequestration. Indeed, as shown in Figure 5, our analyses according to baseline TAV size revealed that a significantly higher risk for coronary obstruction was identified in patients with smaller SE TAVs. In this scenario, the results of the multivariable analysis are reasonable, indicating that STJ diameter, rather than STJ height, is independently associated with coronary obstruction risk in the SE TAV group.

We should cite herein the recently published paper reported by Ochiai et al^20^ for discussion. That study, assessing CT data from a total of 418 post-TAVR patients (258 treated with BE TAVs and 160 treated with SE TAVs), showed the potential risk for coronary obstruction due to SOV sequestration at 10.5% for the BE TAV and 48.8% for the SE TAV, which is also remarkably lower compared with our larger study. The discrepancy between the results of these 2 studies among the Japanese population may be attributed to the following 3 factors. First, the definitions of potential SOV sequestration used are not entirely consistent. In the former study, risk for SOV sequestration was defined as TAV neoskirt level above the STJ or horizontal distance between the first TAV and STJ <2.0 mm, whereas in our study, it was defined as TAV commissure level located above the STJ and TAV-to-STJ distance <2.0 mm in each coronary sinus. It should be taken into consideration that the subtle heterogeneity of definitions may lead to considerably variable rates of potential SOV sequestration.

Second, an extremely high TAV position has been indicated since 2018 at our institution to mitigate the risk for post-TAVR conduction disturbances.^21^ Indeed, in the present study, TAV implantation depth was as small as 2.1 mm in the BE TAV group and 3.3 mm in the SE TAV group. These higher TAV positions, compared with those in previous studies, apparently contributed to the increased incidence of TAV commissure level above the STJ, resulting in a higher risk for SOV sequestration. The trade-off relationship between pacemaker risk after the first TAVR and SOV sequestration risk in future redo TAVR should be central to our daily discussion of young and healthy candidates for TAVR.

Third, a larger valve with underfilling was frequently used in BE TAV implantation for border zone annulus in our study population to obtain a larger effective orifice area, as previously shown.^16^^,^^22^ Generally, by adjusting the filling volume of BE TAVs with limited sizes, continuously distributed native annular areas are covered. However, because the BE TAV has the structural property of foreshortening with TAV expansion, a larger valve size with underfilling demonstrated a significantly greater TAV height than a smaller valve size with overfilling, albeit with comparable TAV areas. For example, according to the manufacturer’s sizing guide, the frame heights of 23- and 26-mm TAVs are 18 and 20 mm, respectively, whereas the actual frame heights of an overfilled 23-mm TAV and an underfilled 26-mm TAV are probably <18 mm and more than 20 mm, respectively. Therefore, in looking ahead to the future redo TAVR procedure, we should also consider the option of a smaller valve with overfilling for patients with long life expectancy.

With its expanding indication to patients with a lower surgical risk profile and longer life expectancy, the demand for redo TAVR will undoubtedly increase in the near future. Redo TAVR simulated by CT imaging is more frequently unfeasible in Asians, who generally have smaller aortic complexes than Europeans and Americans. Although the surgical resection of prosthetic valve leaflets under direct vision during redo TAVR has been proposed as a feasible alternative to minimize the trauma of TAV explantation,^23^ it may not be beneficial for high-risk patients, because of the requirement for general anesthesia, ministernotomy, and cardiac arrest. In contrast, the transcatheter BASILICA technique is a less invasive therapeutic option that can be indicated for such patients. However, intentional laceration of the first TAV leaflets using the BASILICA technique before redo TAVR is theoretically not a solution for numerous patients, because of commissural malalignment and insufficient leaflet splay of the longer leaflets of modern TAVs.^24^ Indeed, our study showed that the BASILICA technique for patients at risk for coronary obstruction by redo TAVR was likely to be unfeasible in 6.5% for the LCA and 5.3% for the RCA in the BE TAV group and in 45.0% for the LCA and 46.7% for the RCA in the SE TAV group. Hence, we should carefully screen for the risk for coronary obstruction using CT imaging before redo TAVR by evaluating the positional relationship between the first TAV and aortic complex structures. It is also important to consider the potential feasibility of future redo TAVR when determining the type, size, and position of the first TAV, while as for the TAV technology, the development of a TAV with a lower commissure height than offered by currently available TAVs is warranted as the first TAV for those patients to mitigate the risk of coronary obstruction in future redo TAVR.

### Study limitations

First and most important, the study was based on a simulation of CT data of patients undergoing TAVR; accordingly, predicting the risk for future redo TAVR is not an exact science. Although SOV sequestration was considered equal to coronary obstruction in our analysis, as in prior simulation studies, no literature is available as yet to support the assumption in real-world practice. Whether adequate coronary perfusion would be provided by blood flow into the SOV around the tube graft created by the displaced leaflets of the first TAV is not strictly determinable, and the coronary obstruction risk may be overestimated. Therefore, the predictive accuracy of our criteria to identify the risk for coronary obstruction in redo TAVR should be validated with a real-world, robust registry of patients undergoing redo TAVR. In addition, our registry had included patients who were deemed at high risk for surgery. Thus, further research focusing on younger patients with lower surgical risk, who are more likely than older, higher risk patients to require redo TAVR, is also warranted.

Second, we excluded patients who did not undergo post-TAVR CT imaging because of impaired renal function and those who had poor-quality CT images, causing a selection bias, which should be taken into account.

Third, our analysis focused only on the SAPIEN 3 and Evolut R and Evolut PRO TAVs implanted at a single center, and these findings cannot be extrapolated to other types of TAVs, although the challenge of redo TAVR is shared by all commercially available TAVs.

Finally, most patients whose CT images were used for our simulation of redo TAVR did not actually experience structural valve deterioration requiring invasive treatment. Although structural abnormalities of TAVs, including degenerative changes, endothelialization, prolapse, and thrombus, potentially affect the risk for coronary obstruction in redo TAVR, they were not assessed in the present study.

## Conclusions

The present CT-based simulation study of redo TAVR demonstrated the potential risk FOR coronary obstruction due to SOV sequestration in a substantial number of Asian patients who received either the BE TAV or the SE TAV. This finding suggests the importance of considering in advance the structural feasibility of future redo TAVR when determining the type, size, and position of the first TAV, especially in Asian candidates for TAVR with long life expectancy.

Perspectives **COMPETENCY IN MEDICAL KNOWLEDGE:** This CT simulation study of redo TAVR in the first SAPIEN 3 and Evolut R and Evolut PRO demonstrated a substantially higher rate of the potential risk for coronary obstruction due to SOV sequestration in Asians with small and low STJs compared with previous studies among Europeans and Americans.

**TRANSLATIONAL OUTLOOK:** It might be relevant not only to screen carefully for the risk for SOV sequestration before actual redo TAVR for Asians but also to consider in advance the structural feasibility of future redo TAVR when determining the first TAV, especially in Asian candidates for TAVR with long life expectancy.

## Funding Support and Author Disclosures

Dr Shirai is the proctor of transfemoral TAVR for Edwards Lifesciences, Medtronic, and Abbott Medical. All other authors have reported that they have no relationships relevant to the contents of this paper to disclose.
