# Supplementary material for: Assessing Potential Risks of Future Redo Transcatheter Aortic Valve Replacement in Asian Patients
Source: JACC Asia. 2023 Oct 31;4(1):25–39. doi: 10.1016/j.jacasi.2023.09.004 (PMC10782404; doi:10.1016/j.jacasi.2023.09.004)
Supplement: Supplemental Appendix [file mmc1.docx]

**SUPPLEMENTAL APPENDIX**

**Assessing Potential Risks of Future Redo Transcatheter Aortic Valve Implantation in Asian Patients**

Norihisa Miyawaki, MD, Kenichi Ishizu, MD, Shinichi Shirai, MD, Kenji Nakano, MD, Tadatomo Fukushima, MD, Euihong Ko, MD, Yasuo Tsuru, MD^,^ Hiroaki Tashiro, MD, Miho Nakamura, MD, Hiroyuki Tabata, MD, Toru Morofuji, MD, Takashi Morinaga, MD, Masaomi Hayashi, MD, Akihiro Isotani, MD, Nobuhisa Ohno, MD, Shinichi Kakumoto, MD,^c^ Kenji Ando, MD


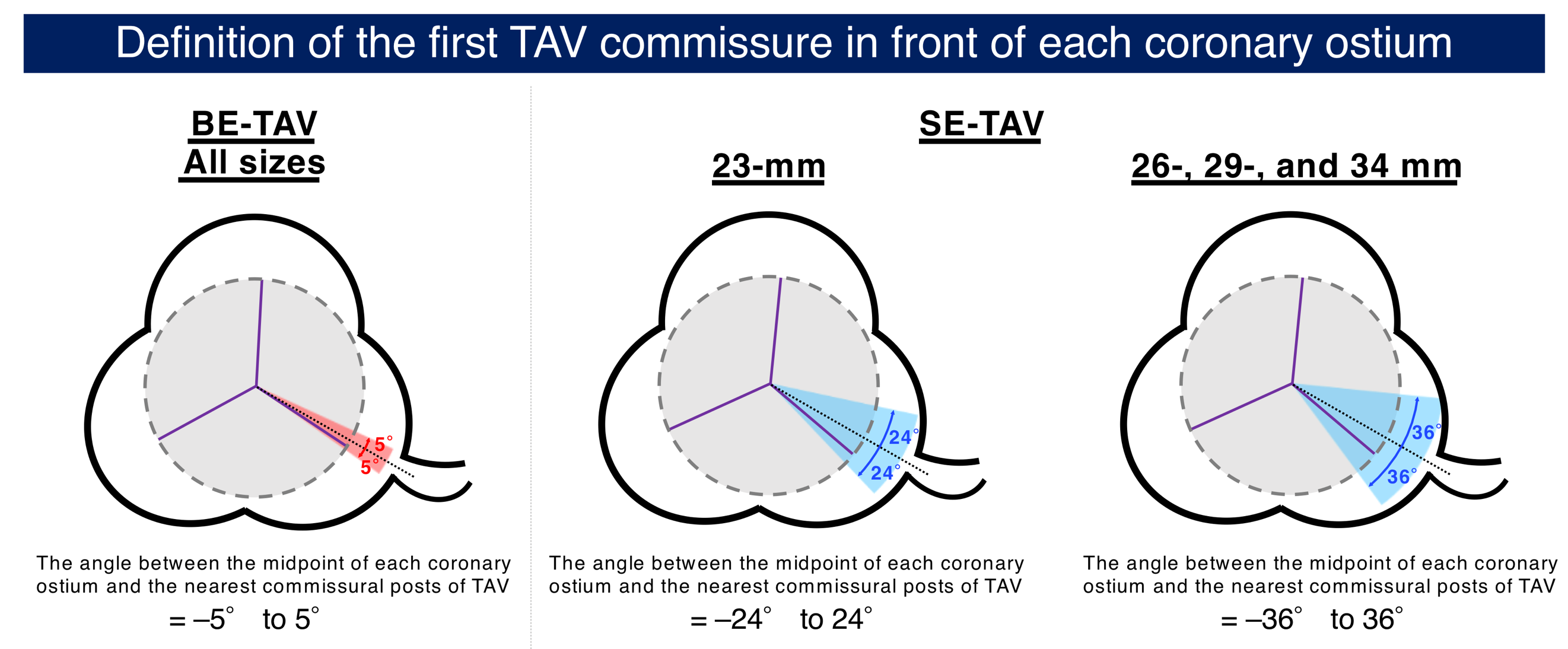
**Supplemental Figure 1. Assessment of TAV Commissural Malalignment**

The angle between the TAV commissure and each coronary ostium was measured using the short-axis view of TAV on post-TAVI CT. The TAV commissure in front of each coronary ostium in our study was defined as shown. BE-TAV = balloon-expandable transcatheter aortic valve; CT = computed tomography; SE-TAV = self-expandable transcatheter aortic valve implantation; TAV = transcatheter aortic valve; TAVI = transcatheter aortic valve implantation.


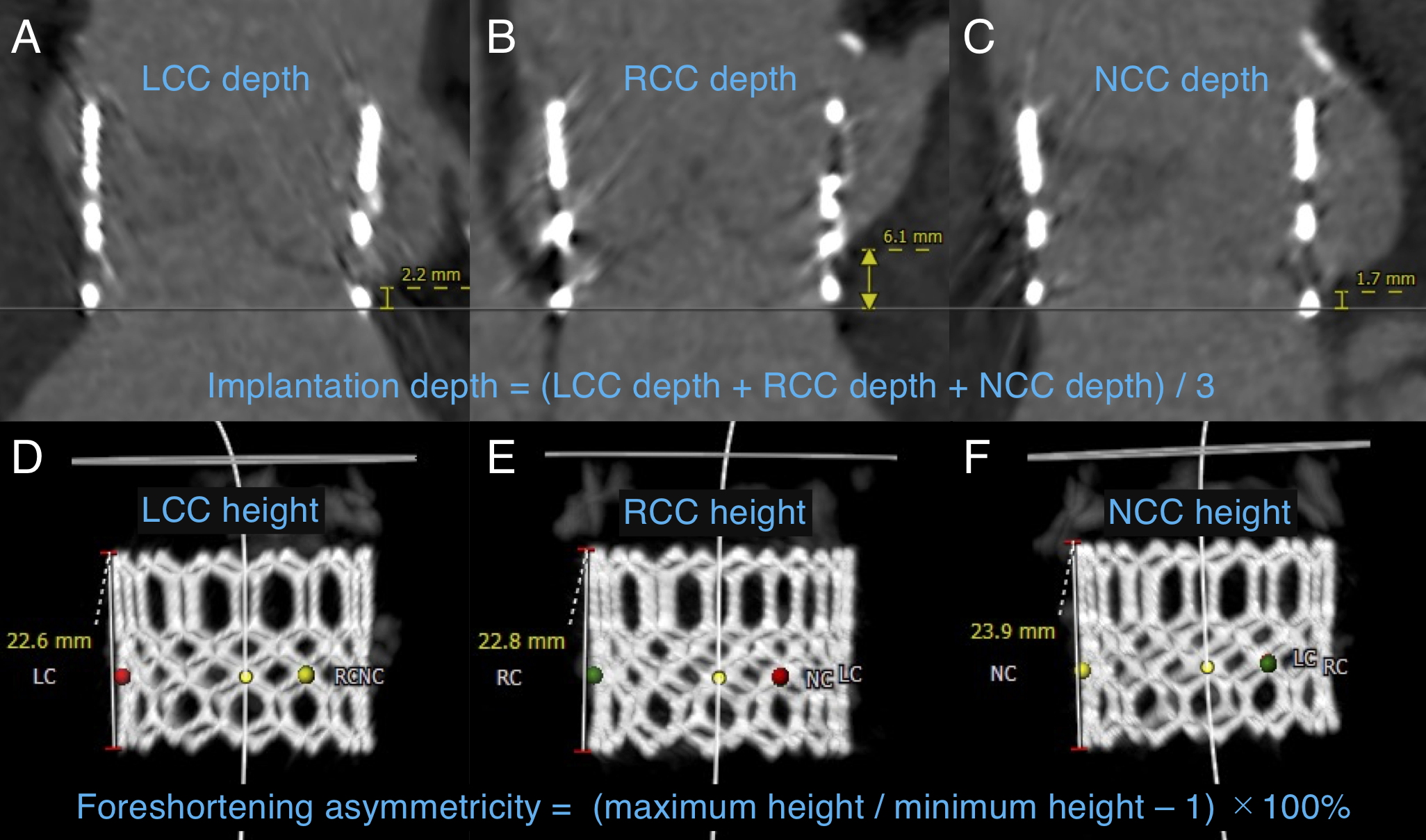
**Supplemental Figure 2. Definition of Implantation Depth and Foreshortening Asymmetricity of TAV**

(A-C) The implantation depth was defined as the mean TAV depth on each cusp above the inflow level. (D-F) The TAV height on each cusp was assessed with maximum intensity projection. The foreshortening asymmetricity of TAV was calculated using the following formula: (maximum TAV height / minimum TAV height – 1) × 100%. LCC = left coronary cusp; NCC = non-coronary cusp; RCC = right coronary cusp; TAV = transcatheter aortic valve.


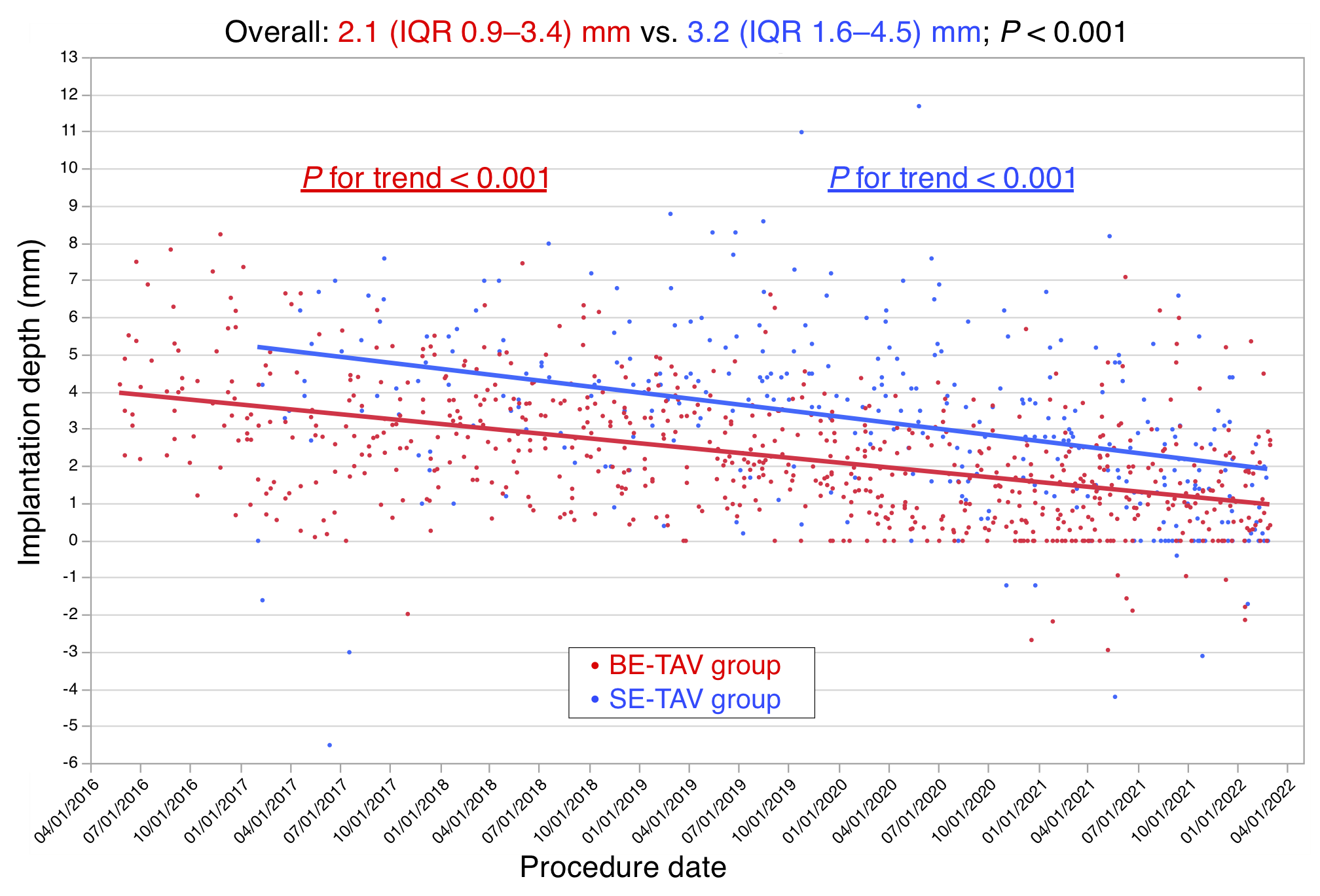
**Supplemental Figure 3. Temporal Trends of Implantation Depth**

Filled circles indicate individual patients according to the date of the procedure and the final implantation depth in millimeters. Red and blue lines represent the regression lines of temporal trends of implantation depth in the BE-TAV group and the SE-TAV group, respectively. BE-TAV = balloon-expandable transcatheter aortic valve; SE-TAV = self-expandable aortic valve.


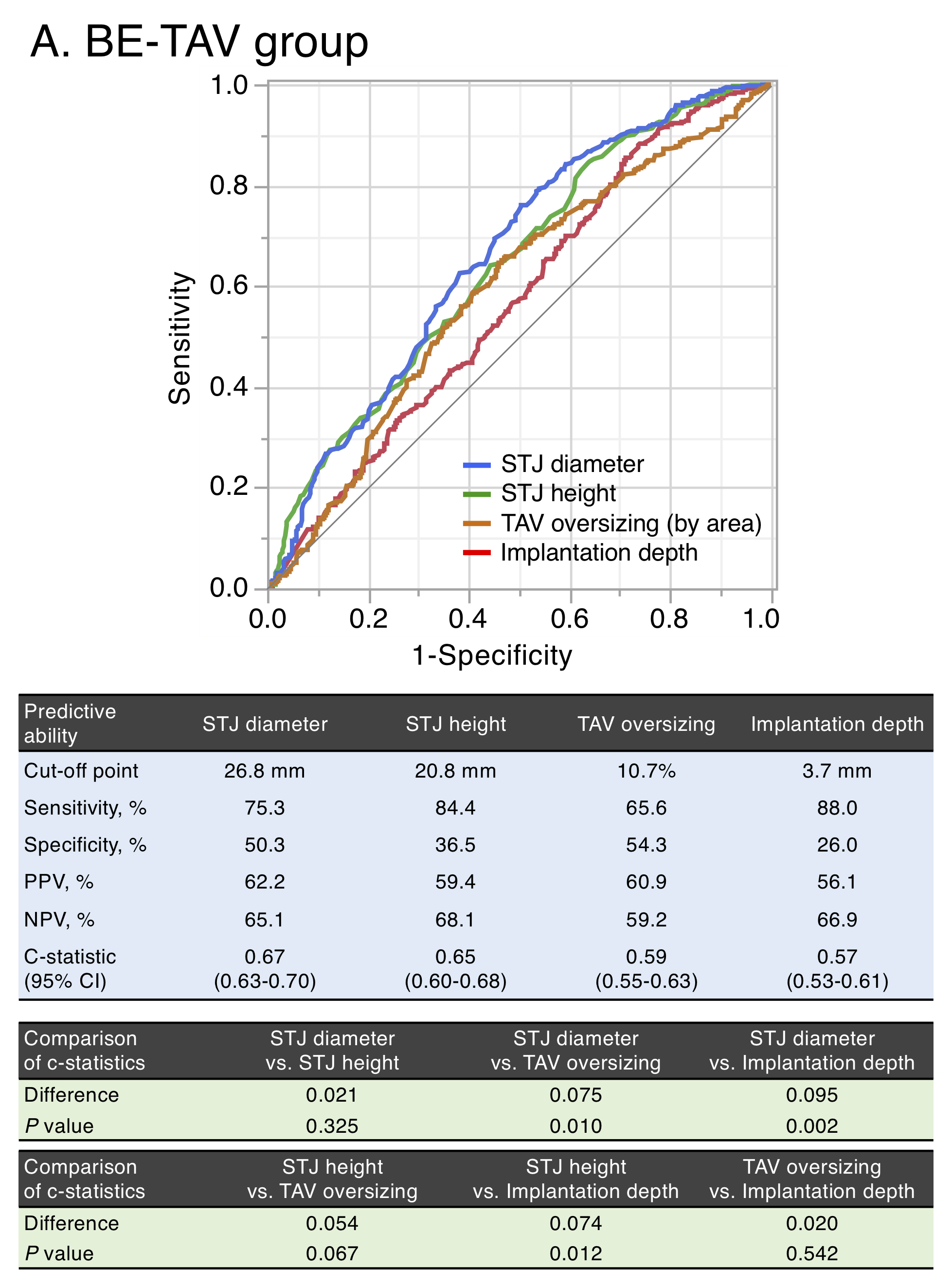

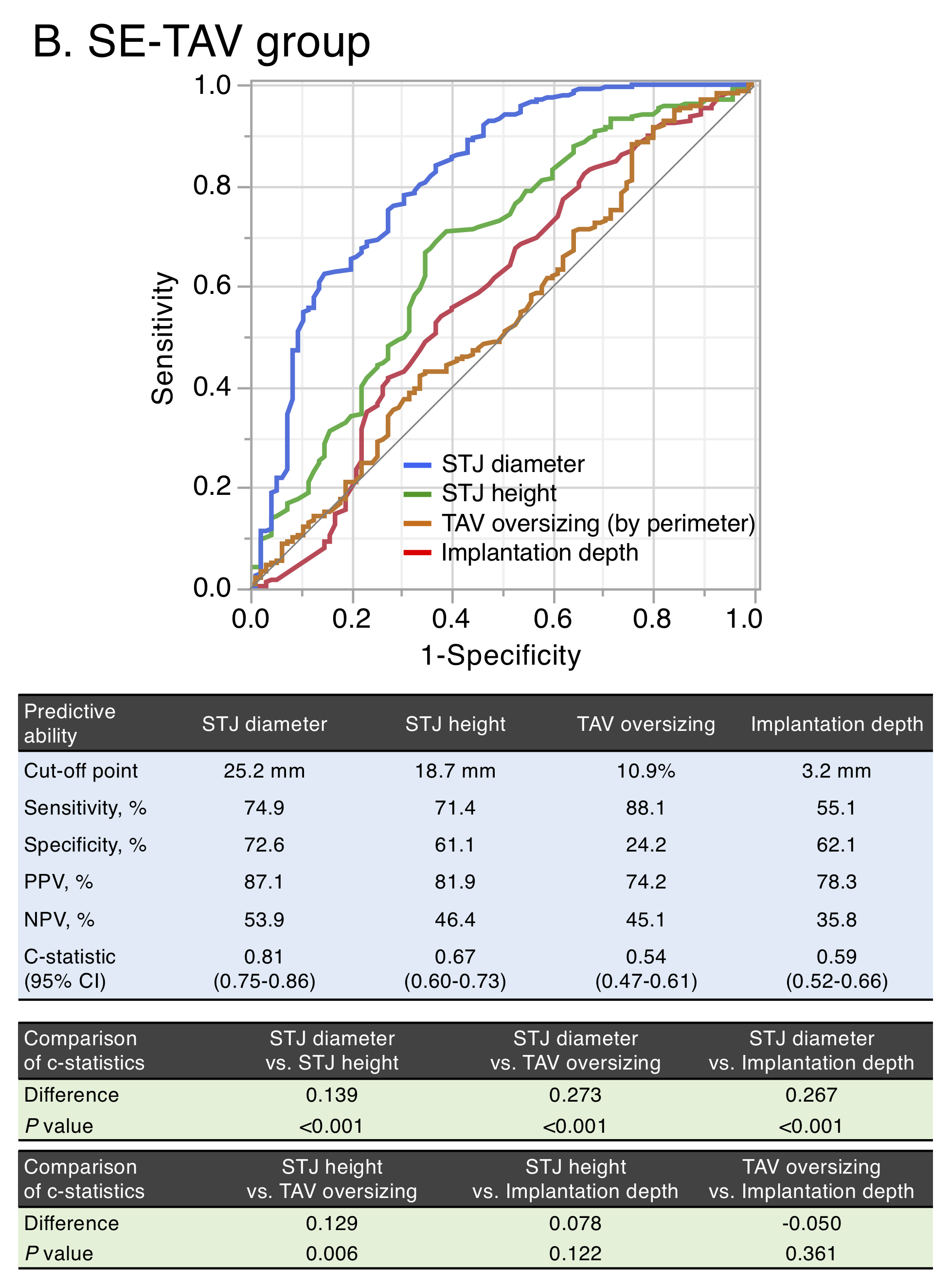
**Supplemental Figure 4. Discrimination Ability of Each Variable for SOV Sequestration**

Receiver operating characteristic (ROC) curves with comparative analyses of the discrimination of each variable for the SOV sequestration in (A) BE-TAV group and (B) SE-TAV group. C-statistic indicates area under the ROC curve for each variable.

BE-TAV = balloon-expandable transcatheter aortic valve; CI = confidence interval; NPV = negative predictive value; PPV = positive predictive value; SE-TAV = self-expandable transcatheter aortic valve; SOV = sinus of Valsalva; TAV = transcatheter aortic valve.

**Supplement Table 1. Baseline Patient Characteristics According to the TAV type**

|  | **Total**  **(n=1084)** | **BE-TAV**  **(n=753)** | **SE-TAV**  **(n=331)** | ***P***  **value** |
| --- | --- | --- | --- | --- |
| **Demographics** |  |  |  |  |
| Age, years | 85 (81–88) | 85 (81–88) | 85 (81–88) | 0.146 |
| Male | 372 (34.3) | 294 (39.0) | 78 (23.6) | <0.001 |
| Height, cm | 149.4 (144.0–157.6) | 150.0 (144.9–159.0) | 148.0 (143.0–154.4) | <0.001 |
| Weight, kg | 50.0 (43.1–58.5) | 51.2 (43.7–59.5) | 47.3 (41.9–54.9) | <0.001 |
| Body mass index, kg/m^2^ | 22.1 (19.9–24.6) | 22.4 (20.0–24.8) | 21.6 (19.5–24.3) | 0.012 |
| Body surface area, m^2^ | 1.4 (1.3–1.6) | 1.4 (1.3–1.6) | 1.4 (1.3–1.5) | <0.001 |
| Clinical Frailty Scale | 3 (3–4) | 3 (3–4) | 3 (3–4) | 0.059 |
| NYHA functional class Ⅲ/Ⅳ | 382 (35.2) | 280 (37.2) | 102 (30.8) | 0.042 |
| STS-PROM score, % | 5.1 (3.6–7.7) | 5.1 (3.5–7.8) | 5.1 (3.6–7.4) | 0.617 |
| **Comorbidities** |  |  |  |  |
| Hypertension | 880 (81.2) | 614 (81.5) | 266 (80.4) | 0.649 |
| Dyslipidemia | 563 (51.9) | 399 (53.0) | 164 (49.6) | 0.296 |
| Diabetes mellitus | 250 (24.1) | 183 (24.3) | 67 (21.3) | 0.042 |
| Atrial fibrillation | 229 (21.1) | 172 (22.8) | 57 (17.2) | 0.034 |
| Coronary artery disease | 409 (37.7) | 299 (39.7) | 110 (33.2) | 0.042 |
| Previous percutaneous coronary intervention | 207 (19.1) | 155 (20.6) | 52 (15.7) | 0.057 |
| Previous valve surgery | 5 (0.5) | 5 (0.7) | 0 (0) | 0.056 |
| Previous permanent pacemaker | 54 (5.0) | 43 (5.8) | 11 (3.3) | 0.008 |
| Peripheral artery disease | 78 (7.2) | 64 (8.5) | 14 (4.2) | 0.009 |
| Chronic obstructive pulmonary disease | 75 (6.9) | 58 (7.7) | 17 (5.1) | 0.116 |
| Cerebrovascular disease | 130 (12.0) | 97 (12.9) | 33 (10.0) | 0.168 |
| Active cancer | 72 (6.6) | 56 (7.4) | 16 (4.8) | 0.103 |
| **Blood tests** |  |  |  |  |
| Hemoglobin, g/dl | 11.4 (10.2–12.5) | 11.5 (10.2–12.6) | 11.2 (10.1–12.2) | 0.034 |
| eGFR, ml/min/1.73 m^2^ | 53.5 (41.1–66.0) | 52.7 (40.9–65.6) | 54.3 (43.2–67.5) | 0.038 |
| Albumin, g/dl | 3.7 (3.4–4.0) | 3.7 (3.4–4.0) | 3.8 (3.4–4.0) | 0.444 |
| Brain natriuretic peptide, pg/ml | 139.1 (61.3–352.1) | 135.3 (60.9–345.6) | 146.8 (62.4–361.8) | 0.577 |
| **Echocardiographic data** |  |  |  |  |
| Aortic valve area, cm^2^ | 0.69 (0.58–0.79) | 0.72 (0.61–0.81) | 0.63 (0.51–0.73) | <0.001 |
| Indexed aortic valve area, cm^2/^m^2^ | 0.50 (0.40–0.55) | 0.50 (0.40–0.57) | 0.44 (0.39–0.50) | <0.001 |
| Mean aortic gradient, mmHg | 44.2 (33.9–57.6) | 40.7 (31.1–52.4) | 53.9 (41.5–68.0) | <0.001 |
| Left ventricular ejection fraction, % | 62.5 (56.4–65.4) | 62.2 (54.6–65.1) | 63.6 (59.5–66.0) | <0.001 |
| Left ventricular end-diastolic diameter, mm | 44.0 (40.2–47.7) | 44.6 (40.8–48.6) | 42.5 (39.1–46.1) | <0.001 |
| Aortic regurgitation ≥ moderate | 59 (5.4) | 47 (6.2) | 12 (3.6) | 0.070 |
| Mitral regurgitation ≥ moderate | 59 (5.4) | 42 (5.6) | 17 (5.1) | 0.767 |
| Tricuspid regurgitation ≥ moderate | 41 (3.8) | 30 (4.0) | 11 (3.3) | 0.595 |
| Systolic pulmonary arterial pressure, mmHg | 31.0 (26.0-37.0) | 31.0 (26.0-37.0) | 31.0 (26.0-37.8) | 0.806 |
| **Pre-TAVI CT data** |  |  |  |  |
| Annulus area, mm^2^ | 408.2 (364.3–464.7) | 420.1 (373.4–481.0) | 387.0 (345.5–432.5) | <0.001 |
| Annulus perimeter, mm | 72.3 (68.2–77.2) | 73.2 (69.2–78.3) | 70.4 (66.3–74.7) | <0.001 |
| LVOT area, mm^2^ | 390.0 (333.1–475.2) | 408.9 (343.6–497.3) | 364.0 (316.3–424.7) | <0.001 |
| STJ height, mm | 18.6 (16.9–20.6) | 19.0 (17.1–20.9) | 18.1 (16.4–19.7) | <0.001 |
| STJ diameter, mm | 25.4 (23.5–27.6) | 25.8 (24.1–28.1) | 24.4 (22.6–26.3) | <0.001 |
| Mean SOV diameter, mm | 29.7 (27.9–31.9) | 30.0 (28.3–32.5) | 28.8 (27.3–30.8) | <0.001 |
| Aorta diameter | 32.5 (30.5–34.4) | 32.8 (30.8–34.6) | 31.7 (30.1–33.5) | <0.001 |
| Left coronary artery height, mm | 13.6 (12.3–15.0) | 13.8 (12.4–15.2) | 13.5 (12.0–14.8) | 0.014 |
| Right coronary artery height, mm | 15.2 (13.1–17.4) | 15.5 (13.5–17.7) | 14.6 (12.6–16.6) | <0.001 |
| Mean STJ/Annulus | 0.061 (0.056–0.067) | 0.061 (0.056–0.067) | 0.063(0.057–0.069) | 0.001 |
| Bicuspid valve | 66 (6.1) | 32 (4.3) | 34 (10.3) | <0.001 |
| **Post-TAVI CT data** |  |  |  |  |
| Implantation depth | 2.4 (1.1–3.8) | 2.1 (0.9–3.4) | 3.2 (1.6–4.5) | <0.001 |

Values are number (percentage) or median (interquartile range). BE-TAV = balloon-expandable transcatheter aortic valve; CT = computed tomography; eGFR = estimated glomerular filtration rate; LVOT = left ventricular outflow tract; NYHA = New York Heart Association; SE-TAV = self-expandable transcatheter aortic valve; SOV = sinus of Valsalva; STJ = sinotubular junction; STS-PROM score = Society of Thoracic Surgeons Predicted risk of Mortality; TAV = transcatheter aortic valve; TAVI = transcatheter aortic valve implantation.
